# Smoker characteristics and trends in tobacco smoking in Rakai, Uganda, 2010–2018

**DOI:** 10.18332/tid/144623

**Published:** 2022-02-28

**Authors:** Fred Nalugoda, Dorean Nabukalu, Joseph Ssekasanvu, Robert Ssekubugu, Connie Hoe, Joseph Kagaayi, Nelson K. Sewankambo, David M. Serwadda, Maria J. Wawer, Kate M. Grabowski, Steven J. Reynolds, Godfrey Kigozi, Ronald H. Gray, Ping T. Yeh, Larry W. Chang

**Affiliations:** 1 Rakai Health Sciences Program, Kalisizo, Uganda; 2 Department of Epidemiology, Johns Hopkins Bloomberg School of Public Health, Johns Hopkins University, Baltimore, United States; 3 Department of International Health, Johns Hopkins Bloomberg School of Public Health, Johns Hopkins University, Baltimore, United States; 4 Heidelberg Institute of Global Health, Heidelberg University Hospital, Heidelberg, Germany; 5 Department of Epidemiology and Biostatistics, School of Public Health, College of Health Sciences, Makerere University, Kampala, Uganda; 6 Department of Medicine, College of Health Sciences, Makerere University, Kampala, Uganda; 7 Department of Disease Control and Environmental Health, School of Public Health, College of Health Sciences, Makerere University, Kampala, Uganda; 8 Department of Pathology, Johns Hopkins School of Medicine, Johns Hopkins University, Baltimore, United States; 9 Division of Infectious Diseases, Department of Medicine, Johns Hopkins School of Medicine, Johns Hopkins University, Baltimore, United States; 10 Division of Intramural Research, National Institute of Allergy and Infectious Diseases, National Institutes of Health, Bethesda, United States

**Keywords:** tobacco, smoking, prevalence, Uganda, Rakai

## Abstract

**INTRODUCTION:**

Tobacco use is a major public health concern, particularly in low- and middle-income countries where 80% of the world’s smokers reside. There is limited population-based data from rural Africa on patterns of tobacco smoking and smoker characteristics. We assessed trends in rates of smoking, characteristics of smokers, and factors associated with smoking using repeat population-based cross-sectional surveys in south-central Uganda.

**METHODS:**

Data accrued over five survey rounds (2010–2018) of the Rakai Community Cohort Study (RCCS) from consenting individuals aged 15–49 years including sociodemographic and behavioral characteristics and smoking status. Proportions of smokers per survey were compared using χ^2^ test for trends, and factors associated with smoking were assessed by multivariable logistic regression.

**RESULTS:**

The prevalence of tobacco smoking in the general population declined from 7.3% in 2010–2011 to 5.1% in 2016–2018, p<0.001. Smoking rates declined among males (13.9–9.2%) and females (2.2–1.8%) from 2010–2011 to 2016–2018. Smoking prevalence was higher among previously married (11.8–11.7%) compared to currently (8.4–5.3%) and never married persons (3.1–1.8%) from 2010–2011 to 2016–2018. Older age (≥35 years) was associated with higher odds of smoking (AOR=8.72; 95% CI: 5.68–13.39 in 2010–2011 and AOR=9.03; 95% CI: 5.42–15.06 in 2016–2018) compared to those aged <35 years (AOR=4.73; 95% CI: 3.15–7.12 in 2010–2011 and AOR=4.83; 95% CI: 2.95–7.91 in 2016–2018). Primary and secondary/higher education level was significantly associated with lower odds of smoking (AOR=0.20; 95% CI: 0.14–0.29 in 2010–2011 and AOR=0.26; 95% CI: 0.18–0.39 in 2016–2018) compared to no education (AOR=0.43; 95% CI: 0.31–0.59 in 2010–2011 and AOR=0.48; 95% CI: 0.34–0.68 in 2016–2018). Number of sexual partners and HIV status were not associated with smoking.

**CONCLUSIONS:**

We observed declining trends in tobacco smoking in the Rakai region of rural Uganda. Smoking was more prevalent in men, older individuals, individuals who were previously married, and individuals with lower education. The decline in smoking may be due to tobacco control efforts, but there is a continued need to target sub-populations with higher smoking prevalence.

## INTRODUCTION

The World Health Organization (WHO) Report on the Global Tobacco pandemic 2021 shows that the prevalence of tobacco smoking among people aged >15 years has decreased from 22.7% to 17.5%^1^. While WHO also indicated that smoking rates have decreased by 6.7% since 2000, they still estimate that over 1 billion people around the world still smoke and predict rapid increase in prevalence among African men^2,3^. WHO has attributed 8 million premature deaths annually worldwide to tobacco smoking.

Uganda, a low-income country in Sub-Saharan Africa, is undergoing rapid population growth, urbanization, and improved survival to older age. Non-communicable diseases are now a major burden of disease in addition to communicable diseases. WHO estimated that about 10% of Uganda’s population (approximately 1.8 million people) smoked in 2010, but data on smoking in the general population is limited. A nationwide survey in 2014 revealed that 7.4% of participants were daily smokers of whom 79.3% were males, and the highest rate of smoking was in those aged 30–49 years^4^. Cross-sectional studies of tobacco smoking among school pupils aged 13–17 years reported a smoking prevalence of 5.3–5.6% in Uganda’s capital city, Kampala^5,6^.

Currently, the surveillance of tobacco use among adults in Uganda is done through the quinquennial Uganda Demographic and Health Surveys (UDHS) and the Global Adult Tobacco Survey^4^. The UDHS 2011 reported the prevalence of daily smoking as 15.7%, higher among men than women and increasing with age^7^. The prevalence of smoking was 14–15% among men and 1–2% in women in rural Uganda^8^. Rural areas have higher smoking rates than urban areas, potentially associated with lower income and education level, and higher unemployment^9^. In addition, tobacco control policies and other regulatory factors often benefit urban areas more than rural areas^10^, and tobacco crops are a source of income for many rural areas; thus, tobacco is more normalized in the rural culture^11^. Tobacco smoking research focusing on rural areas in Africa in addition to enforcing control measures is therefore critically needed.

Given the paucity of data on patterns of tobacco smoking and limited population-based data from rural Africa, our primary aim is to examine trends in the prevalence of tobacco smoking, characteristics of smokers and factors associated with smoking using data collected in 2010–2018 from the Rakai Community Cohort Study (RCCS) in trading and agrarian communities in south-central Uganda.

## METHODS

Data from participants enrolled in RCCS between 2010 and 2018 in trading and agrarian communities was used as repeat cross-sectional surveys for this study. The RCCS is an open, population-based cohort of consenting persons aged 15–49 years surveyed on average every 14–18 months, covering different calendar years in 36 communities in Rakai and neighboring districts of south-central Uganda^12^. The RCCS conducts a household census, followed by an interview of eligible consenting individuals to collect sociodemographic and behavioral data, including a question on whether they currently smoke cigarettes and/or pipes. A venous blood sample is collected for HIV diagnosis at each survey. HIV testing is done in the field using a parallel three rapid test algorithm.

Smoking prevalence was computed and compared using χ^2^ for trends between survey rounds. Factors associated with tobacco smoking were assessed using unadjusted and adjusted odds ratios (AOR) with 95% confidence intervals (CI) using logistic regression.

## RESULTS

**Figure 1:**
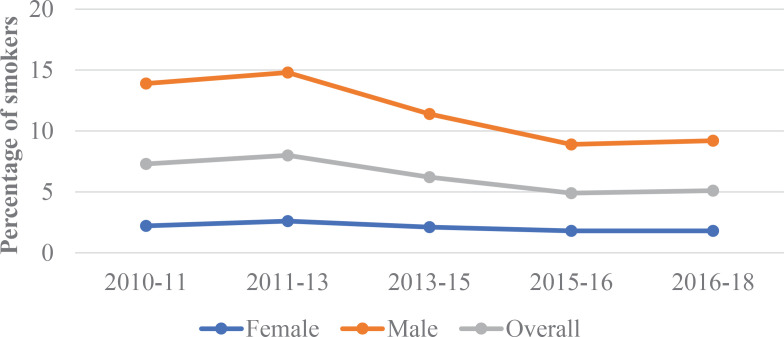
Tobacco smoking trends by survey round and gender

Approximately 9635 to 12500 participants were enrolled per survey round. Prevalence of tobacco smoking by round and by socio-behavioral factors are presented in Table 1. The prevalence of tobacco smoking declined from 7.3% in 2010–2011 to 5.1% in 2016–2018. Smoking prevalence declined in 2015–2016 and remained constant at the most recent time point in 2018, suggesting a plateau (Figure 1). Prevalence of tobacco smoking was significantly higher among men compared to women, 13.9 versus 2.2% (p<0.001) in 2010–2011 and 9.2 versus 1.8% (p<0.001) in 2016–2018 (Table 1). Persons aged ≥35 years had a higher prevalence of tobacco smoking compared to younger age-groups across all survey rounds. Smoking prevalence was higher among the previously married (11.8% and 11.7%) compared to the currently (8.4% and 5.3%) and the never married (3.1% and 1.8%) in 2010–2011 and 2016–2018, respectively. Smoking was more common among those reporting more than one sex partner (13.6% and 8.6%) compared to those with one partner (6.7% and 4.7%) or with no sexual relationship (4.2% and 3.1%) during the same time periods. Smoking was higher among truck drivers (20.2% and 8.2%) compared to other occupations, and in HIV-positive (9.9% and 6.2%) compared to HIV-negative individuals. Smoking prevalence was higher among those with no education (18.4% and 15.1%) compared to those who completed primary (8.9%, 6.3%), or secondary school/higher education (3.6%, 2.5%), in 2010–2011 and 2016–2018, respectively. The observed general decline in tobacco smoking prevalence over time was consistent across most covariates.

**Table 1:** Tobacco smoking prevalence by round and socio-behavioral factors in trading and agrarian communities, 2010–2018

*Characteristics*	*R14 2010–2011 n/N (%)*	*R15 2011–2013 n/N (%)*	*R16 2013–2015 n/N (%)*	*R17 2015–2016 n/N (%)*	*R18 2016–2018 n/N (%)*
**Overall**	704/9635 (7.3)	878/10927 (8.0)	724/11732 (6.2)	616/12496 (4.9)	626/12323 (5.1)
**Sex**					
Male	587/4219 (13.9)	722/4866 (14.8)	589/5168 (11.4)	492/5520 (8.9)	501/5463 (9.2)
Female	117/5416 (2.2)	156/6061 (2.6)	135/6564 (2.1)	124/6976 (1.8)	125/6860 (1.8)
**Age** (years)					
15–24	60/3501 (1.7)	115/4103 (2.8)	62/4533 (1.4)	36/4742 (0.8)	49/4763 (1)
25–34	264/3454 (7.6)	299/3799 (7.9)	232/3773 (6.1)	180/3836 (4.7)	177/3596 (4.9)
35–44	307/2091 (14.7)	366/2399 (15.3)	320/2709 (11.8)	285/3076 (9.3)	284/3047 (9.3)
45–49	73/589 (12.4)	98/626 (15.7)	110/717 (15.3)	115/842 (13.7)	116/917 (12.6)
**Marital status**					
Currently married	467/5535 (8.4)	567/6177 (9.2	441/6406 (6.9)	347/6795 (5.1)	349/6614 (5.3)
Previously married	148/1253 (11.8)	182/1433 (12.7)	191/1606 (11.9)	197/1739 (11.3)	208/1777 (11.7)
Never married	89/2847 (3.1)	129/3317 (3.9)	92/3720 (2.5)	72/3962 (1.8)	69/3932 (1.8)
**HIV status**					
Negative	574/8325 (6.9)	721/9413 (7.7)	585/10189 (5.7)	495/10841 (4.6)	532/10818 (4.9)
Positive	130/1310 (9.9)	157/1514 (10.4)	139/1543 (9)	121/1655 (7.3)	94/1505 (6.2)
**Number of sexual partners** (past 12 months)					
One partner	404/6038 (6.7)	517/6841 (7.6)	359/6636 (5.4)	327/7476 (4.4)	334/7097 (4.7)
More than one	215/1584 (13.6)	249/1800 (13.8)	218/1942 (11.2)	190/2329 (8.2)	205/2378 (8.6)
No sexual relationships	85/2013 (4.2)	112/2286 (4.9)	147/3153 (4.7)	99/2691 (3.7)	87/2848 (3.1)
**Occupation**					
Agriculture/housewife	354/4399 (8)	388/4647 (8.3)	344/4824 (7.1)	280/4881 (5.7)	323/5114 (6.3)
Bar/restaurant	15/205 (7.3)	22/298 (7.4)	17/299 (5.7)	12/302 (4)	11/289 (3.8)
Truck	18/89 (20.2)	34/264 (12.9)	26/248 (10.5)	27/249 (10.8)	30/364 (8.2)
Trade/shop	93/1347 (6.9)	130/1614 (8.1)	102/1704 (6)	84/1875 (4.5)	82/1817 (4.5)
Other	224/3595 (6.2)	304/4104 (7.4)	235/4657 (5)	213/5189 (4.1)	180/4739 (3.8)
**Religion**					
Christian	105/2208 (4.8)	175/2834 (6.2)	125/3178 (3.9)	81/2987 (2.7)	107/2913 (3.7)
Muslim	24/376 (6.4)	23/447 (5.1)	16/581 (2.8)	22/514 (4.3)	23/485 (4.7)
Other	575/7051 (8.2)	680/7646 (8.9)	583/7973 (7.3)	513/8995 (5.7)	496/8925 (5.6)
**Education level**					
None	65/353 (18.4)	73/417 (17.5)	68/395 (17.2)	51/388 (13.1)	56/371 (15.1)
Primary	515/5816 (8.9)	619/6554 (9.4)	531/6973 (7.6)	456/7198 (6.3)	448/7080 (6.3)
Secondary/Higher	124/3466 (3.6)	186/3956 (4.7)	125/4364 (2.9)	109/4910 (2.2)	122/4872 (2.5)

Table 2 shows unadjusted and adjusted logistic regression analyses of odds of smoking by socio-behavioral characteristics in 2010–2011 and 2016–2018. Being female was associated with lower odds of smoking compared to males (AOR=0.10; 95% CI: 0.07–0.12 in 2010–2011 and AOR=0.13; 95% CI: 0.10–0.17 in 2016–2018). The odds of smoking increased with age across all survey rounds. Previously married individuals (AOR=2.04; 95% CI: 1.61–2.59 in 2010–2011 and AOR=3.12; 95% CI: 2.52–3.87 in 2016–2018) had increased odds of smoking compared to those who were currently married. Primary and secondary/higher education level was significantly associated with lower odds of smoking (AOR=0.20; 95% CI: 0.14–0.29 in 2010–2011 and AOR=0.26; 95% CI: 0.18–0.39 in 2016–2018) compared with no education (AOR=0.43; 95% CI: 0.31–0.59 in 2010–2011 and AOR=0.48; 95% CI: 0.34–0.68 in 2016–2018). Multiple sexual partners, HIV status, and religion, were not significantly associated with tobacco smoking.

**Table 2:** Multivariable logistic regression for tobacco smoking prevalence in trading and agrarian communities

*Characteristics*	*Round 14 (2010–2011) OR (95% CI)*	*Round 14 (2010–2011) AOR (95% CI)*	*Round 18 (2016–2018) OR (95% CI)*	*Round 18 (2016–2018) AOR (95% CI)*
**Sex**				
Male (Ref.)	1	1	1	1
Female	0.14 (0.11–0.17)***	0.10 (0.07–0.12)***	0.18 (0.15–0.22)***	0.13 (0.10–0.17)***
**Age** (years)				
15–24 (Ref.)	1	1	1	1
25–34	4.75 (3.75–6.31)***	4.73 (3.15–7.12)***	4.98 (3.62–6.86)***	4.83 (2.95–7.91)***
35–44	9.87 (7.44–13.09)***	8.72 (5.68–13.39)***	9.89 (7.28–13.44)***	9.03 (5.42–15.06)***
45–49	8.11 (5.69–11.56)***	7.22 (4.44–11.76)***	13.93 (9.89–19.62)***	11.48 (6.73–19.61) ***
**Marital status**				
Currently married (Ref.)	1	1	1	1
Previously married	1.45 (1.19–1.77)***	2.04 (1.61–2.59)***	2.38 (1.99–2.85)***	3.12 (2.52–3.87)***
Never married	0.35 (0.28–0.44)***	0.97 (0.68–1.37)	0.32 (0.25–0.42)***	1.02 (0.66–1.57)
**HIV status**				
Negative (Ref.)	1	1	1	1
Positive	1.49 (1.22–1.82)***	1.10 (0.88–1.39)	1.29 (1.03–1.62)*	0.82 (0.63–1.05)
**Number of sexual partners**				
One (Ref.)	1	1	1	1
More than one	2.19 (1.84–2.61)***	0.91 (0.74–1.10)	1.91 (1.60–2.29)***	1.00 (0.82–1.23)
No sexual relationships	0.62 (0.48–0.78)***	0.81 (0.61–1.09)	0.64 (0.50–0.81)***	0.83 (0.62–1.11)
**Occupation**				
Agriculture/housewife (Ref.)	1	1	1	1
Bar/restaurant	0.90 (0.53–1.54)	1.39 (0.79–2.44)	0.59 (0.32–1.08)	0.92 (0.47–1.83)
Truck	2.90 (1.71–4.91)***	1.23 (0.70–2.15)	1.33 (0.90–1.97)	0.71 (0.47–1.09)
Trade/shop	0.85 (0.67–1.07)	0.69 (0.54–0.90)**	0.70 (0.55–0.90)**	0.70 (0.53–0.91)**
Other	0.76 (0.64–0.90)**	0.89 (0.70–2.15)	0.59 (0.49–0.71)***	0.79 (0.63–0.98)*
**Religion**				
Christian (Ref.)	1	1	1	1
Muslim	1.37 (0.86–2.16)	1.22 (0.74–2.01)	1.31 (0.82–2.07)	1.29 (0.76–2.20)
Other	0.78 (1.44–2.20)***	0.81 (0.64–1.03)	1.54 (1.25–1.91)***	0.64 (0.50–0.81)*
**Education level**				
None (Ref.)	1	1	1	1
Primary	0.43 (0.32–0.57)***	0.43 (0.31–0.59)***	0.38 (0.28–0.51)***	0.48 (0.34–0.68)***
Secondary/Higher	0.16 (0.12–0.23)***	0.20 (0.14–0.29)***	0.14 (0.10–0.20)***	0.26 (0.18–0.39)***

AOR: adjusted odds ratio.

* p<0.05

** p<0.01

*** p<0.001.

## DISCUSSION

Our study findings show that tobacco smoking prevalence declined by about 30% between 2010 and 2018. Prevalence of smoking was higher among men than women, was greater among previously married persons, and increased with age in rural south-central Uganda. The declining trend in smoking prevalence is evident through 2015 and plateaued thereafter.

The decline in tobacco smoking prevalence between 2010–2018 is compatible with WHO projections^2^. Findings of higher smoking prevalence among men than women are consistent with other studies conducted among adults in a rural population-based cohort in Uganda^8^, elsewhere in the region, and widely in Sub-Saharan Africa^7,13,14^, and globally^2^. The Ugandan study also showed high smoking prevalence among illiterate residents.

We hypothesize that the decline in the prevalence of tobacco smoking could be attributed to the enacting of tobacco control measures by the Ugandan government. In 2007, Uganda became a Party to the WHO Framework Convention on Tobacco Control (FCTC)^15^. The FCTC mandates that every party to the treaty adopt policies such as smoking bans, health warnings, and promotion, advertising and sponsorship bans. The country progressively implemented these policies^16^, overlapping with this study period. Taxes and price increases are considered the most impactful and powerful tool for reducing tobacco use, and it is estimated that a tax increase which raises tobacco prices by 10% can decrease consumption by as much as 8% in LMICs^17^. In Uganda, between 2004 and 2011, the excise tax on tobacco was increased from 0% in 2005–2006 to 5.3% in 2007–2009, followed by a further increase of 10% in 2011–2012^18^. In subsequent years, tobacco taxes fluctuated between 8–10% until 2017–2018 after the amendment of the 2017 excise duty Act^19^. The excise tax on tobacco and amendments of the excise duty Acts resulted in increased tobacco prices, which, together with other policies, laws and regulations may have contributed to decreased tobacco consumption observed in our study. These are encouraging findings since the decline in tobacco smoking over time is likely to contribute to a reduction in tobacco-attributable diseases such as lung and heart diseases, chronic respiratory diseases, cancers, and diabetes^1^. It is important for the government to intervene by enforcing existing tobacco smoking control guidelines and regulations, as well as targeting sub-populations with high prevalence, and illiterate residents in rural areas.

### Strengths and imitations

The study’s strength is that it uses data from communities in rural Uganda, where growth of leaf tobacco as an economic activity is likely to happen. The ability for the study to comprehensively measure tobacco use was limited since only one question was asked at each survey; questions about other methods of tobacco use, such as tobacco chewing and snuff, were not asked. Questions on type of tobacco use (e.g. commercial or home grown), or quantity and duration of smoking, were not asked. However, studies on agricultural practices in similar rural settings in Uganda show that more than 30% of households grow some tobacco, largely for personal consumption or local sale^20^, and should also be targeted for control interventions. Tobacco smoking is therefore likely to be higher than what this study suggests. In addition, Uganda, as the rest of Sub-Saharan Africa has experienced rapid population grown over the years contributing to a large denominator of the population surveyed in this study. This may have also contributed to the decline in the proportions of smokers at the different time points.

## CONCLUSIONS

The decreasing trend of tobacco smoking in this study should not deter further tobacco control and prevention interventions. Rather, our findings could help inform intervention programs targeting sub-populations with higher smoking prevalence, especially in rural areas. Such interventions include community engagement highlighting deleterious health effects of tobacco use, peer education programs on benefits of not smoking since tobacco use is partly peer pressure driven, and identification and engagement of change agents from within targeted sub-populations with messages that prevent tobacco smoking. It is crucial that involvement of the local community administrative structure to ensure appropriate implementation of the interventions and adhering to control guidelines to prevent smoking is underscored.

Future research should focus on comprehensive assessment of types of tobacco consumption including cigarettes, pipes, chewing, and snuff, and whether tobacco is locally grown or commercially purchased, and on passive smoking to determine the magnitude of tobacco use.

## Data Availability

The data supporting this research are available from the authors on reasonable request.
